# The impact of task complexity and self-regulated supports on cognitive load and emotional engagement through multimodal analysis in the metaverse learning environment

**DOI:** 10.3389/fpsyg.2026.1805065

**Published:** 2026-04-15

**Authors:** Eunbyul Yang, Jeeheon Ryu, Haram Choi, Inki Kim

**Affiliations:** 1 Department of Elementary Education, Jeju National University, Jeju, Republic of Korea; 2 Institute of Educational Research Department of Education, College of Education, Chonnam National University, Gwangju, Republic of Korea; 3 The Grainger College of Engineering, University of Illinois Urbana-Champaign, Urbana, IL, United States

**Keywords:** cognitive load, emotional engagement, metaverse learning environment, multimodal analysis, self-regulated learning, task complexity

## Abstract

This study examined how task complexity and self-regulated learning (SRL) support strategies relate to learners’ cognitive load and emotional engagement in a Metaverse Learning Environment (MLE) using multimodal indicators. College students (*N* = 42) completed three game-embedded tasks of low, mid, and high complexity while receiving cognitive-regulated support (CS), motivational-regulated support (MS), or no additional support (control group, CG). Learners’ cognitive and emotional engagement were assessed using self-reported mental effort and multimodal measures for process approaches, an EEG-derived mental workload index (beta/[alpha + theta]), frontal alpha asymmetry (FAA), and facial expression analysis (FEA) metrics of task-facing orientation and engagement. Task complexity showed robust but non-monotonic effects across modalities. The mid-complexity task elicited higher EEG workload and emotional engagement than the low- and high-complexity tasks, whereas task-facing orientation was lower in the high-complexity task than in the low- and mid-complexity tasks. FAA was also higher in the mid-complexity task than in the high-complexity task. SRL support strategies were modality-specific: the control group reported higher mental effort than the CS group, and the CS group showed higher expressive engagement than both the MS and CG groups during the mid-complexity task. These findings suggest that cognitive support may reduce subjective strain while sustaining emotional involvement under moderate challenge. More broadly, the results highlight the value of multimodal measurement for distinguishing perceived effort from physiological and behavioral indicators of learner experience in immersive learning environments.

## 1 Introduction

Cognitive Load Theory (CLT) explains learning outcomes in terms of how task complexity and instructional design features impose demands on learners’ limited working-memory resources (Sweller et al., 1998). CLT has provided instructional principles to help learners maintain optimal learning loads despite a range of learning difficulties. Recently, as virtual interactive learning environments such as the metaverse have been adopted across various learning contexts, CLT has empowered instructional design to provide focused attention and facilitate engagement in immersive learning. In regard to the interactive learning process, Self-Regulated Learning (SRL) has improved the use of limited cognitive resources, creating trade-offs between support and additional processing demands in complex interactive environments (Seufert, 2018). Prior research has examined whether cognitively oriented or motivationally oriented regulation is more effective for helping learners manage limited cognitive resources in complex learning environments. Some studies suggest that cognitively focused scaffolds can reduce unnecessary processing by guiding attention and strategy use during task performance. Other work emphasizes that motivational support is equally important because learners’ task value, persistence, and self-efficacy influence how effectively those resources are mobilized. In immersive environments, however, it remains unclear whether cognitive or motivational support is more effective for reducing perceived strain while sustaining productive engagement.

Recent research on interactive learning suggests that cognitive load and emotional engagement are dynamically associated in Metaverse Learning Environments (MLEs). MLEs can promote active cognitive involvement, but they also require learners to distribute attention across multiple concurrent task demands during learning. The Cognitive Affective Model of Immersive Learning (CAMIL) proposes that immersive affordances such as presence and agency shape both cognitive and affective factors, including cognitive load, interest, motivation, and self-regulation (Makransky and Petersen, 2021). Empirical studies likewise indicate that immersive environments can heighten enjoyment, interest, and engagement, but they can also increase extraneous cognitive load or affective distraction when novelty, interface demands, or poorly guided interactivity consume learners’ limited working-memory resources (Parong and Mayer, 2021; Petersen et al., 2022). From this perspective, emotional engagement should not be treated as separate from cognitive processing; rather, it may support focused attention and sustained effort when learners experience sufficient control and task value, or co-occur with overload when environmental demands exceed regulatory capacity (Dubovi, 2022). This relationship is especially relevant to SRL because scaffolds that guide monitoring and strategy use may reduce the subjective cost of regulation while helping learners remain emotionally absorbed in the task (Hsu and Lee, 2026). To design an efficient MLE requires an integrated perspective that examines how cognitive load and emotional engagement co-evolve across the range of task complexities rather than assuming that either construct alone fully captures the learning experience.

A central issue in CLT research concerns not only how to design for cognitive load but also how to measure load validity as learning unfolds in complex, interactive environments (Klepsch et al., 2017). Although self-report instruments remain useful, they are typically administered retrospectively and therefore provide coarse summaries that may not capture moment-to-moment fluctuations in processing during learning (Klepsch et al., 2017; Leppink et al., 2013). Although CAMIL has shown the relational structure among the factors, it does not ensure a multimodal perspective. Accordingly, recent work in CLT and learning analytics has emphasized multimodal measurement approaches that combine synchronized physiological and behavioral indicators (Larmuseau et al., 2020). Crucially, relying on a single modality may obscure the distinction between the learner’s perceived effort and their actual processing intensity. Therefore, examining cognitive load and emotional engagement across multiple channels is essential to understanding the efficiency of learning processes under concurrent task demands. In this study, we examine whether multimodal measures, including EEG and facial-expression data, can capture the ongoing dynamics of learners’ cognitive processing and emotional engagement during task performance.

### 1.1 Cognitive load theory in metaverse learning environments

Metaverse Learning Environments (MLEs) are networked, persistent 3D virtual spaces where learners interact through avatars and engage with learning tasks embedded in simulated environments (Damaševičius and Sidekerskienė, 2024). MLEs offer rich interaction and learner autonomy, which can support engagement, but they also increase the likelihood that learners must distribute attention across multiple concurrent streams of information and action (Kaddoura and Al Husseiny, 2023). In game-based MLEs, learners must coordinate domain learning with goal-directed gameplay and spatial exploration, which can shift when and where cognitive demands peak during learning episodes (Tsai et al., 2022). Because immersive interfaces often contain visually salient elements that are not instructionally relevant, instructional supports are beneficial only when they are designed to guide attention without competing for it or introducing redundant processing (Seufert, 2018).

Against this backdrop, CLT provides a useful framework for explaining how these environment- and design-induced demands interact with learners’ limited working-memory resources (Sweller et al., 1998). Within CLT, cognitive load is commonly discussed in terms of intrinsic and extraneous sources of demand: intrinsic load is driven by the element interactivity inherent in the material, whereas extraneous load arises from how information and activities are presented. In immersive environments, this distinction is consequential because interface management and interaction requirements (e.g., navigation and visually dense displays) can introduce additional processing demands beyond those required by the learning content itself (Makransky and Petersen, 2021).

### 1.2 Task complexity as an intrinsic load manipulation in MLEs

In MLEs, complexity effects can be amplified because learners must process task-relevant information while simultaneously managing interface and interaction demands (e.g., navigation, avatar control, and visually dense displays), which can add extraneous processing demands beyond the learning content itself (Makransky and Petersen, 2021; Seufert, 2018). Task complexity is a primary determinant of cognitive load because increasing task demands typically increases element interactivity and the amount of information that must be coordinated in working memory; within Cognitive Load Theory, these complexity-driven demands are mainly interpreted as intrinsic sources of load (Sweller et al., 1998; Van Merriënboer and Sweller, 2010). However, in interactive learning contexts, load indicators may not vary strictly linearly with intended complexity, because learners’ effort allocation and regulation can shift when tasks are experienced as under-challenging, optimally challenging, or overwhelming (Seufert, 2018).

Prior work in game- and simulation-based learning suggests that metacognitive prompting can support learners’ strategic monitoring and task performance during complex activities, although its benefits depend on how it is embedded in ongoing gameplay (Castronovo et al., 2022; Verpoorten et al., 2014; Zumbach et al., 2020). Taken together, these points motivate examining task complexity in MLEs not only as an intrinsic-load manipulation, but also as a context in which instructional supports may simultaneously assist regulation and alter extraneous processing demands depending on their delivery characteristics. This raises a design question: supports intended to help regulation may also compete for attention and add extraneous demands, particularly when delivered as on-screen prompts during gameplay.

### 1.3 Self-regulated learning and emotional engagement

In MLEs, learners often exercise substantial autonomy as they progress through tasks embedded in interactive environments. Under such conditions, self-regulated learning (SRL)—the goal-directed regulation of cognitive, motivational, and behavioral processes—becomes particularly relevant because learners must plan, monitor, and evaluate their learning while managing task progression and interaction demands (Pintrich and De Groot, 1990; Pintrich, 2000). Pintrich’s framework highlights that regulation is not limited to cognitive strategy use but also includes motivational and emotional regulation, suggesting that SRL supports may shape both perceived effort and learners’ emotional–motivational involvement during ongoing activity (Pintrich, 2000). A common design approach is to embed SRL scaffolds as prompts that cue learners to monitor understanding, revisit content, or sustain task value and self-efficacy. Prior work in serious games and simulation-based learning indicates that such metacognitive prompts can improve strategic monitoring and learning outcomes, although effects depend on when and how prompts are delivered during gameplay (Verpoorten et al., 2014; Zumbach et al., 2020; Castronovo et al., 2022).

From a CLT perspective, SRL prompts can function in two competing ways: they may reduce extraneous load by directing attention and supporting effective strategy selection, or they may increase extraneous load if they are poorly timed or intrusive (Seufert, 2018; Wang 2025a; Wang 2025b). This dichotomy highlights the importance of evaluating SRL supports not merely by whether they reduce absolute load, but by their instructional efficiency (Paas and van Merriënboer, 1993)—the degree to which supports optimize the ratio between invested mental effort and performance or engagement. In visually dense environments, pop-up style prompts may act as attentional interruptions; however, if they successfully scaffold regulation, they may lower the subjective cost of processing while maintaining high engagement. Thus, in MLEs, it is useful to examine whether different SRL prompt types are associated with distinct patterns of efficiency regarding cognitive load and emotional engagement.

Cognitive load and emotional engagement are analytically distinct but dynamically intertwined in immersive learning environments (Makransky and Petersen, 2021). In this study, emotional engagement is conceptualized as a dynamic process of emotional and motivational fluctuations rather than a final learning outcome. Specifically, in an immersive environment, emotional engagement serves as a critical indicator of how learners monitor and regulate their psychological states in response to task demands. By capturing real-time physiological and behavioral proxies, we aim to operationalize emotional engagement as the immediate motivational orientation and expressive activation that sustains the self-regulated learning process (Dindar et al., 2020; Li et al., 2024).

In MLEs, learners must coordinate content processing with navigation, interaction, and self-regulation, so changes in task demand can simultaneously shape perceived effort and emotional–motivational involvement. When demands are well matched to learners’ regulatory capacity, challenge may sustain focused engagement even under relatively high processing demands; when demands exceed that capacity, the same conditions may produce subjective strain and disengagement. Accordingly, understanding learner experience in MLEs requires examining not only how much mental effort learners invest, but also how that effort co-evolves with emotional engagement across task episodes (Makransky and Lilleholt, 2018).

### 1.4 Multimodal data approach and study contribution

In immersive, game-based MLEs, learners’ cognitive demands and emotional–motivational states can fluctuate across task episodes as they coordinate content processing with interaction and navigation. This creates a measurement challenge for CLT- and SRL-oriented research: although self-report instruments remain useful, they are typically administered retrospectively and thus provide relatively coarse summaries of perceived effort (Leppink et al., 2013; Ouwehand et al., 2021).

Accordingly, recent work in learning analytics and cognitive load research has emphasized multimodal measurement, in which synchronized subjective, physiological, and behavioral channels are integrated to capture learning processes as they unfold over time (Molenaar et al., 2023). In this framework, each modality provides a complementary signature. However, rather than simply seeking convergence, modern multimodal research posits that divergences between subjective and physiological measures can provide deeper insights into the learning experience (Azevedo and Gašević, 2019). For instance, a learner might report low effort (appraisal) while maintaining high physiological arousal (process), indicating highly efficient processing. Building on this approach, the present study adopts a synchronized multimodal design in an MLE to examine whether task complexity and SRL support condition show convergent or modality-specific patterns across channels. Concretely, we combine subjective ratings with EEG-derived indices and facial-video-derived behavioral metrics, time-aligned to task episodes, to better characterize how cognitive load and emotional engagement evolve during gameplay and how SRL prompt types relate to these dynamics.

**Figure 1:**
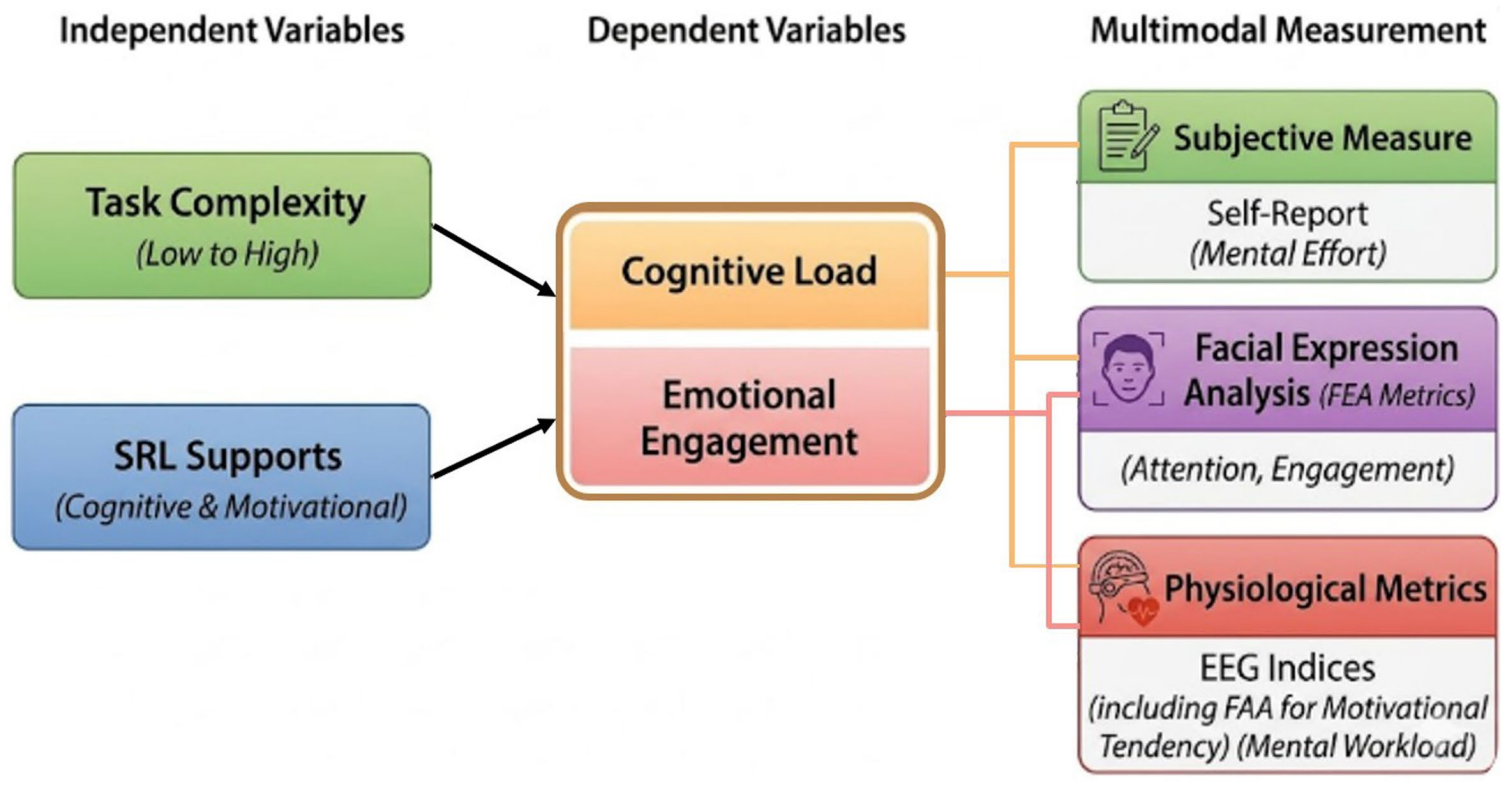
Conceptual research model illustrating the effects of task complexity and SRL supports on cognitive load and emotional engagement, assessed using a multimodal measurement framework.

Figure 1 shows the conceptual framework of this study. Task complexity (low, mid, high) and self-regulated learning strategies (cognitive support, motivational support, no support) serve as independent variables that influence learners’ cognitive load and emotional engagement during gameplay in a metaverse learning environment. The dependent variables are assessed using a multimodal measurement framework that integrates subjective measures (self-reported mental effort), physiological metrics (EEG-derived mental workload), and behavioral–emotional indicators derived from facial expression analysis (FEA) and frontal alpha asymmetry (FAA).

### 1.5 Research aims and questions

This study investigates how the type of SRL support strategy and task complexity relate to learners’ cognitive load and emotional engagement during gameplay in a Metaverse Learning Environment. We compare cognitive-regulated support, motivational-regulated support, and a control condition, using multimodal indicators to capture temporal variation in learners’ mental effort, attention, affect, and engagement during tasks of differing complexity. The following research questions guide the study:

- RQ1: How do SRL support strategy type and task complexity affect multimodal indicators of cognitive load in an MLE?
- RQ2: How do SRL support strategy type and task complexity affect multimodal indicators of emotional engagement in an MLE?

## 2 Methods

### 2.1 Participants and research design

Forty-two college students participated in the study (13 men, 32.1%; 29 women, 67.9%; *M* = 23.33, *SD* = 2.45). Participants were randomly assigned to one of three SRL support conditions: cognitive-regulated support (CS; *n* = 13), motivational-regulated support (MS; *n* = 14), or a control condition (CG; *n* = 15). CS received prompts targeting cognitive learning strategies in the MLE, MS received prompts targeting motivational regulation strategies, and CG received only task-navigation instructions (i.e., no SRL prompts). The dependent variables were cognitive load and emotional engagement measured via self-report, FEA, and EEG. Participants received monetary compensation. The study was approved by the university’s institutional review board (No. 1040198-220204-HR-008-03).

### 2.2 Educational game design

#### 2.2.1 Self-regulated learning gamified design

The independent variable was SRL support condition (CS vs. MS vs. CG). We implemented two SRL prompt types: cognitive-regulated prompts (CS) and motivational-regulated prompts (MS), drawing on SRL frameworks that distinguish cognitive and motivational regulation processes (Pintrich, 2000). CS prompts targeted cognitive strategy regulation (e.g., monitoring understanding, managing pace, and revisiting content), whereas MS prompts targeted motivational regulation (e.g., mastery orientation, self-efficacy, and task value; Cennamo and Ross, 2000; Schwinger et al., 2007; Yang, 2000). The control condition (CG) received only task-navigation/game progress instructions without SRL-support content.

SRL prompts were implemented as fixed, pre-scripted message sets: each condition (CS, MS, CG) had a predefined set of messages, and all participants within a given condition received the same message content. Prompts were delivered as pop-up windows overlaid on the metaverse platform and were triggered at standardized time points (at the beginning of the game, after the lecture video, and after each learning task). In total, participants encountered prompts seven times throughout gameplay.

Table 1 maps the CS and MS prompt content onto Zimmerman’s (2002) three-phase SRL cycle (forethought, performance, and self-reflection). In the forethought phase, CS prompts oriented learners to the upcoming content and supported planning, whereas MS prompts emphasized goal setting and motivational preparation. During the performance phase, CS prompts supported monitoring and strategy use, while MS prompts emphasized task value and encouragement. During the self-reflection phase, CS prompts guided evaluation of correctness and strategy use, whereas MS prompts supported appraisal of task value and motivational reflection.

**Table 1:** Mapping of SRL support strategies to learning phases and game-based activities.

Learning phase		Activities	Cognitive support	Motivational support
Forethought	Activation	Goal presentation	Providing an overview of learning	Setting performance goals
	Demonstration	Presenting learning content	Checking the pace of learning materials	Perceiving task value
Performance	Application	Practice (low level) understanding task	Reconfirming content	Enhancing intellectual competence
		Practice (mid-level) applying task	Comprehension check	Performance approach self-instruction
	Integration	Practice (high level) problem-solving task	Checking problem-solving pace	Enhancing personal meaning
Self-reflection	Reflection	Assessment	Checking incorrect answers	Perceiving achievement value

For the detailed instructional design of the learning experience, the educational game was organized into five stages following gamification-based SRL design principles (Qiao et al., 2022) and aligned with Merrill (2002) instructional principles: (1) activation, (2) demonstration, (3) application, (4) integration, and (5) reflection. These stages describe the sequence of learning activities and game progression within the MLE.

#### 2.2.2 Game scenario

**Figure 2:**
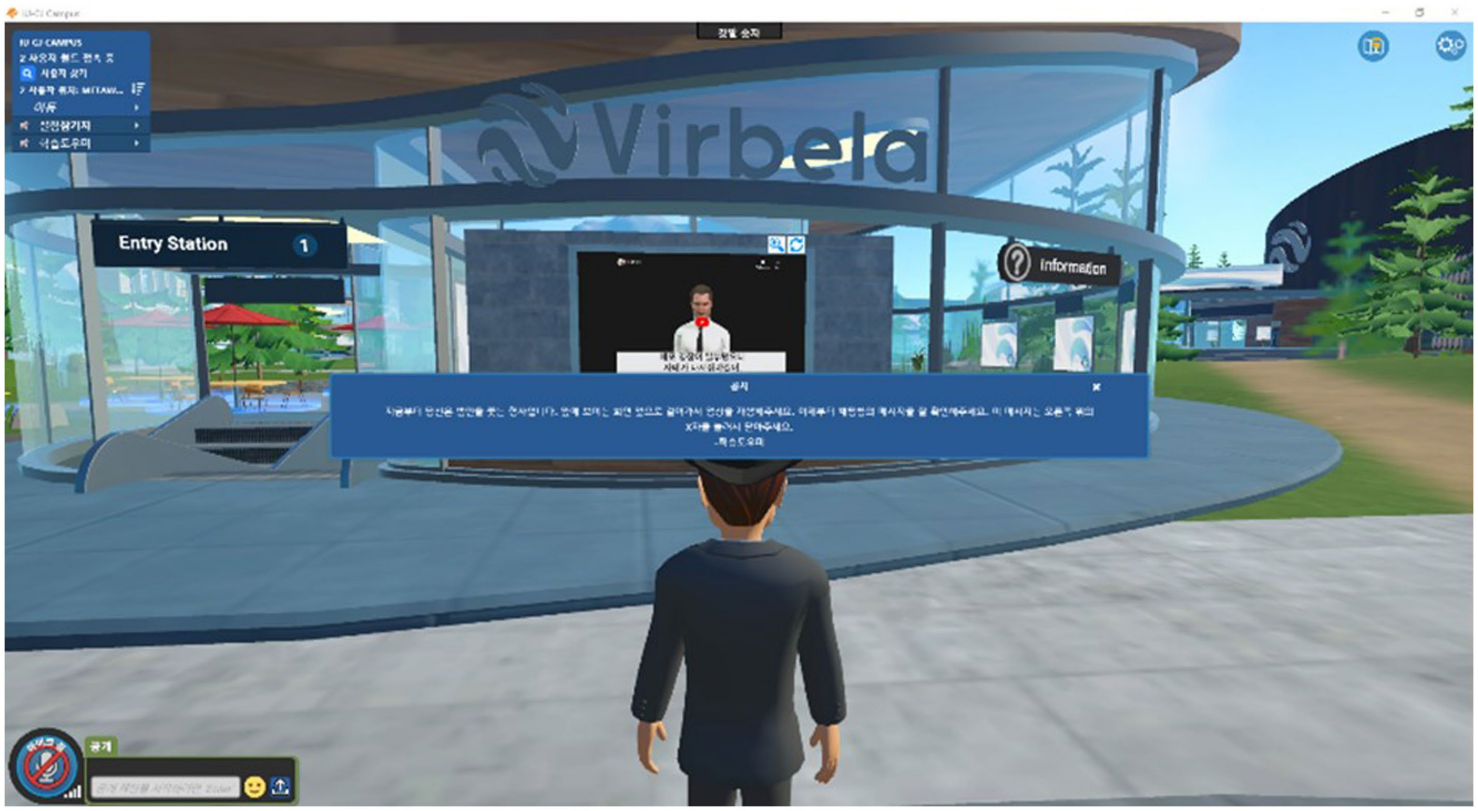
SRL support prompt examples in the Metaverse learning environment.

We developed an educational game in the Metaverse platform Virbela (eXp World Technologies LCC, CA, US; Figure 2). Participants controlled virtual avatars to navigate an island and assumed the role of a detective. The overarching mission was to locate and arrest a suspect hidden on the island by following game rules, progressing through learning activities, and collecting hints. Participants entered and exited different locations on the island and first viewed a video lecture on economic investment strategies for college students. After the lecture, participants completed three learning tasks situated in different locations within the MLE.

#### 2.2.3 Task complexity in the learning task

Task complexity was manipulated across three learning tasks (low, mid, high), guided by Bloom’s taxonomy as an instructional design rationale. Complexity in this study was operationalized to encompass both the intrinsic difficulty of the content and the modality-specific processing demands (e.g., immediate calculation vs. self-paced reading) required by the task format. The low-complexity task focused on conceptual understanding: participants accessed short reading materials presenting key concepts and studied them independently in the MLE. Each reading set was presented as a single slide, enabling self-paced navigation and review. The mid-complexity task required mental arithmetic: participants were given a return-rate formula and calculated investment returns under specified conditions using mental calculation. The high-complexity task involved open-ended problem solving: participants read real-world investment failure cases, studied relevant principles, and wrote proposed solutions to the presented problems. All participants completed the three tasks in a fixed sequence (low → mid → high). After each task, participants completed a brief quiz to assess understanding. After completing all tasks, the operator provided a final hint to support completion of the mission.

### 2.3 Data collection

#### 2.3.1 Cognitive loads

We collected data through self-reported mental effort, FEA, and EEG to characterize learners’ cognitive processing during gameplay. Mental effort was measured via self-report, EEG frequency-band metrics were used to estimate momentary mental workload, and the FEA-derived attention metric was used as a behavioral proxy of attentional allocation during task engagement. Consistent with multi-method approaches in CLT research, these indicators were treated as complementary proxies rather than interchangeable measures of a single latent construct, and we did not assume a one-to-one mapping between any indicator and a specific load type (Paas, 1992; Paas and van Merriënboer, 1994).

##### 2.3.1.1 Mental effort by self-reports

To assess mental effort, this study utilized a questionnaire originally developed by Ryu (2011). This variable is predicated on the hypothesis that increased learner dedication to learning outcomes or problem-solving correlates with heightened mental effort. Consequently, a higher degree of mental effort by the learner suggests a greater cognitive load required for learning. Thus, mental effort is treated as a subjective indicator of invested cognitive effort during task processing, which may reflect learning-relevant processing but is not assumed to map uniquely onto a single load type. Mental effort was measured using four items, including “I exerted significant effort to solve the evaluation questions presented” and “I focused my mental effort while reading the evaluation questions.” This questionnaire’s internal consistency index was recorded at 0.77.

##### 2.3.1.2 Attention by FEA

We recorded participants’ facial expressions in real time using a webcam on the monitor. We also used Affectiva’s AFFDEX, the FEA algorithm from iMotions (iMotions, Copenhagen, Denmark), to analyze facial expressions. AFFDEX is a computer-vision-based facial analysis toolkit that estimates facial landmarks, head pose, and facial action units, and derives higher-level emotional metrics such as attention and engagement (Bishay et al., 2023; McDuff et al., 2016). We used the AFFDEX attention metric as a behavioral proxy for attentional allocation, not as a direct measure of cognitive load. In iMotions/AFFDEX, attention is primarily derived from head pose (forward-facing orientation) and is expressed on a 0–100 scale (Otamendi and Sutil Martín, 2020). Therefore, higher attention values are interpreted as stronger task-facing orientation during the task segment, whereas lower values may reflect off-screen orientation or reduced alignment with the display. Attention was included as a behavioral proxy indicating task-facing orientation during each episode, supporting cross-channel triangulation of task engagement (Rueda et al., 2023). In the present study, attention was used to complement self-report and EEG indices by indicating when participants were behaviorally oriented toward task-relevant information during the tasks, thereby supporting cross-channel triangulation of task engagement.

##### 2.3.1.3 Mental workload by EEG

Electroencephalography (EEG) data were collected using a wireless EEG system (Enobio 8, Neuroelectrics, Barcelona, Spain). The system was equipped with electrodes positioned according to the international 10–20 system at prefrontal sites (F7, AF7, Fp1, Fz, Fp2, AF8, F8), along with an additional reference electrode. Prefrontal electrode sites were selected because of their relevance to cognitive processing during learning tasks.

EEG signals were recorded continuously throughout the gameplay session at a sampling rate of 60 Hz. Prior to data collection, conductive gel was applied between the electrodes and scalp to improve signal quality. Signal quality was monitored during the experiment using the iMotions software. Participants were instructed to minimize head movements during the experiment to facilitate stable EEG and facial expression recording.

EEG data were processed using the iMotions 9.3 Biometric Research Platform, with additional processing conducted using R. After data collection, EEG signals were analyzed in the frequency domain using power spectral density estimates. Mean spectral power values were calculated for the following frequency bands, which are commonly used in cognitive workload research and are compatible with the sampling rate of the EEG system: Delta: 1–3 Hz, Theta: 4–8 Hz, Alpha: 8–12 Hz, Beta: 12–30 Hz. Only frequency bands up to 30 Hz were analyzed due to the sampling rate constraints of the EEG system. No gamma-band activity was included in the analyses.

Multiple EEG frequency bands have been proposed as an indicator of cognitive load. Prior studies have integrated established EEG power bands that correlate with cognitive workload. These bands––theta, alpha, and beta––correspond to different workload aspects, such as attention, vigilance, and mental fatigue (Fernandez Rojas et al., 2020). Mental effort was assessed using an EEG-derived index based on the ratio of beta power to the sum of alpha and theta power, which has been used in prior studies as an indicator of cognitive workload during task performance (Smit et al., 2005). For each participant and task segment, mean power values for the beta, alpha, and theta bands were calculated across the selected prefrontal electrodes. The resulting values were used as indicators of relative mental effort during the learning tasks and were entered into the statistical analyses. The mental effort index was computed using the following equation:


$$\text{Mental Workload}=\frac{B\mathrm{eta}\;\text{power}}{A\text{lpha power}+\text{Theta power}}$$

In line with a multi-method CLT measurement approach, we interpret the indicators used in this study as complementary rather than interchangeable. Self-reported mental effort reflects participants’ perceived investment in task processing and is commonly used as a subjective indicator of load. The EEG beta/(alpha + theta) index is treated as a physiological indicator of mental workload during task performance. In contrast, the facial-expression-derived attention metric is interpreted as a proxy for attentional allocation rather than a direct measure of cognitive load. Together, these measures allow examination of convergence and divergence in load-related processes across subjective, physiological, and behavioral channels. Importantly, this design does not claim to isolate intrinsic vs. extraneous vs. germane load with separate instruments; instead, it tests whether experimental manipulations produce convergent or dissociable patterns across channels during task episodes (Klepsch et al., 2017; Paas and van Merriënboer, 1994).

#### 2.3.2 Emotional engagement

Both FEA and EEG data were employed to characterize learners’ emotional responses during gameplay. The facial-expression-based metric was emotional engagement (AFFDEX/iMotions), and frontal alpha asymmetry (FAA) was computed from EEG as an index related to motivational direction (van Bochove et al., 2016). Dimensional approaches conceptualize emotional states along separable dimensions (e.g., arousal and valence), and such frameworks have been widely used to describe emotions during interactive media experiences. However, in multimodal measurement, the key issue is how each indicator maps onto the underlying construct. In the present study, the FEA-derived engagement metric is treated as a proxy for expressive activation, whereas FAA is interpreted as an index of approach–withdrawal-related motivational tendency rather than a direct measure of valence. This dual-indicator approach allows examination of whether task episodes that elicit stronger expressive activation also show convergent changes in EEG-based motivational direction.

##### 2.3.2.1 Engagement by FEA

Engagement refers to learners’ emotional involvement during learning activities (Fredricks et al., 2004). Using the AFFDEX/iMotions pipeline described above, we used the engagement metric as a behavioral proxy for emotional involvement during each task episode. Engagement is operationalized as a composite measure of facial muscle activation (expressive intensity) derived from multiple action units and reported on a 0–100 scale (Affectiva, 2017). Because automated facial metrics can be sensitive to tracking quality and do not map perfectly onto internal emotional states, we interpret engagement as outward expressiveness rather than a direct measure of arousal or discrete emotions and therefore applied transparent QC and episode-level aggregation procedures (Stöckli et al., 2018).

##### 2.3.2.2 Frontal alpha asymmetry by EEG

Frontal alpha asymmetry (FAA) is commonly used as an EEG-derived index related to emotional–motivational tendencies. Because alpha power is inversely related to cortical activity, FAA is typically computed as the log ratio of right-to-left frontal alpha power. In the present study, FAA was interpreted within an approach–withdrawal framework: higher values indicate relatively greater approach-related motivational tendency, whereas lower values indicate relatively greater withdrawal-related tendency; importantly, FAA should not be treated as a direct proxy of emotional valence, and its interpretation can vary by task context and preprocessing choices (Allen et al., 2018; Fischer et al., 2018). FAA scores were computed from alpha-band power at F7 and F8 as:


$$\text{Frontal Alpha Asymmetry}\;(\mathrm{FAA})=\ln(\frac{\alpha (F8)}{\alpha (F7)})$$

### 2.4 Procedure

**Figure 3:**
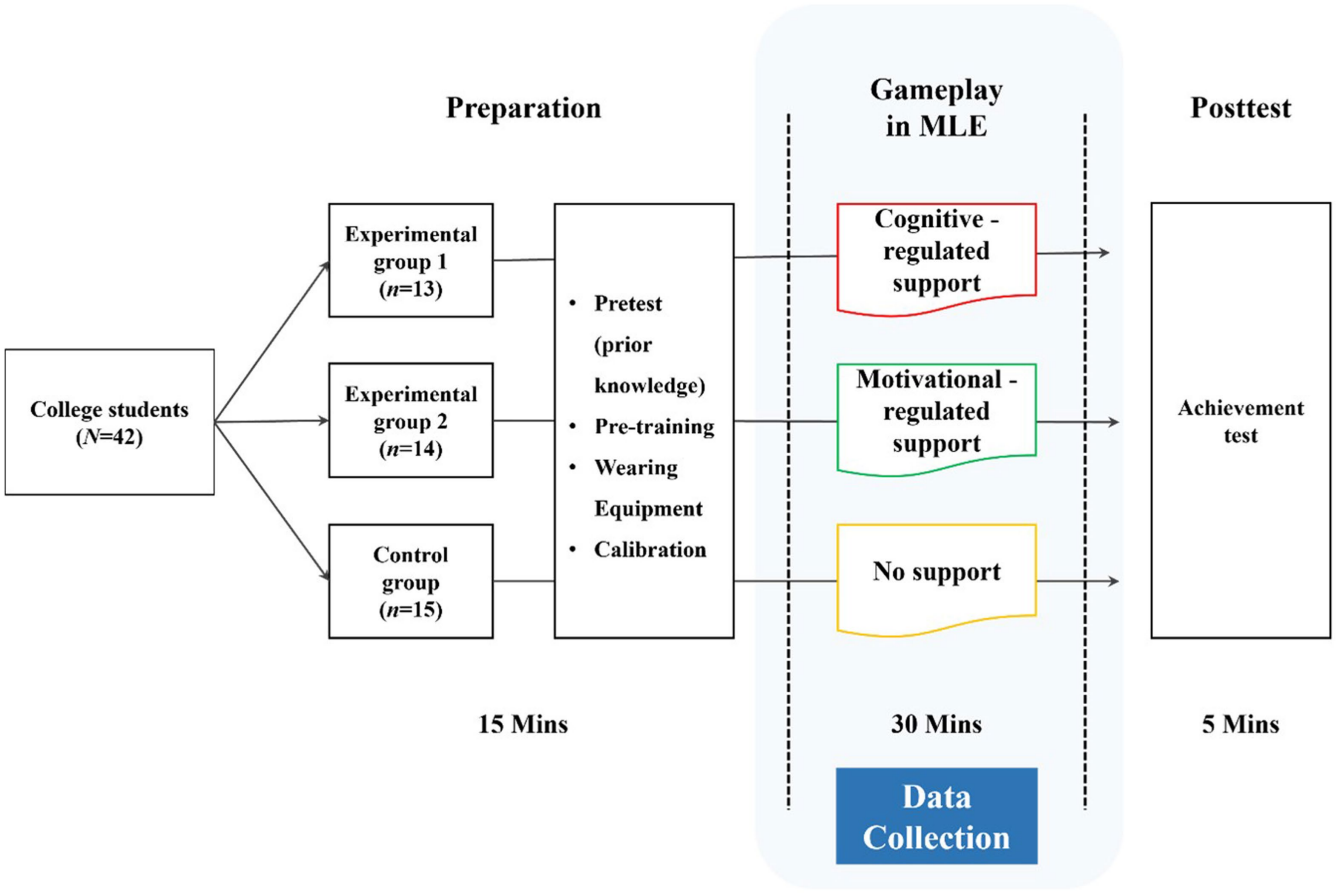
Experimental procedure.

The experiment consisted of two phases (Figure 3). In the preparation phase, participants completed a brief screening and practice session to familiarize themselves with avatar navigation and the MLE interface, followed by calibration for multimodal recording. In the educational game phase, participants navigated the metaverse space, viewed the lecture video, and completed the three learning tasks (low, mid, high). Biometric data (EEG and facial video) were recorded continuously during the game phase and later segmented into task-specific time windows for analysis. The total session lasted approximately 50 min, including preparation.

### 2.5 Data analysis

**Figure 4:**
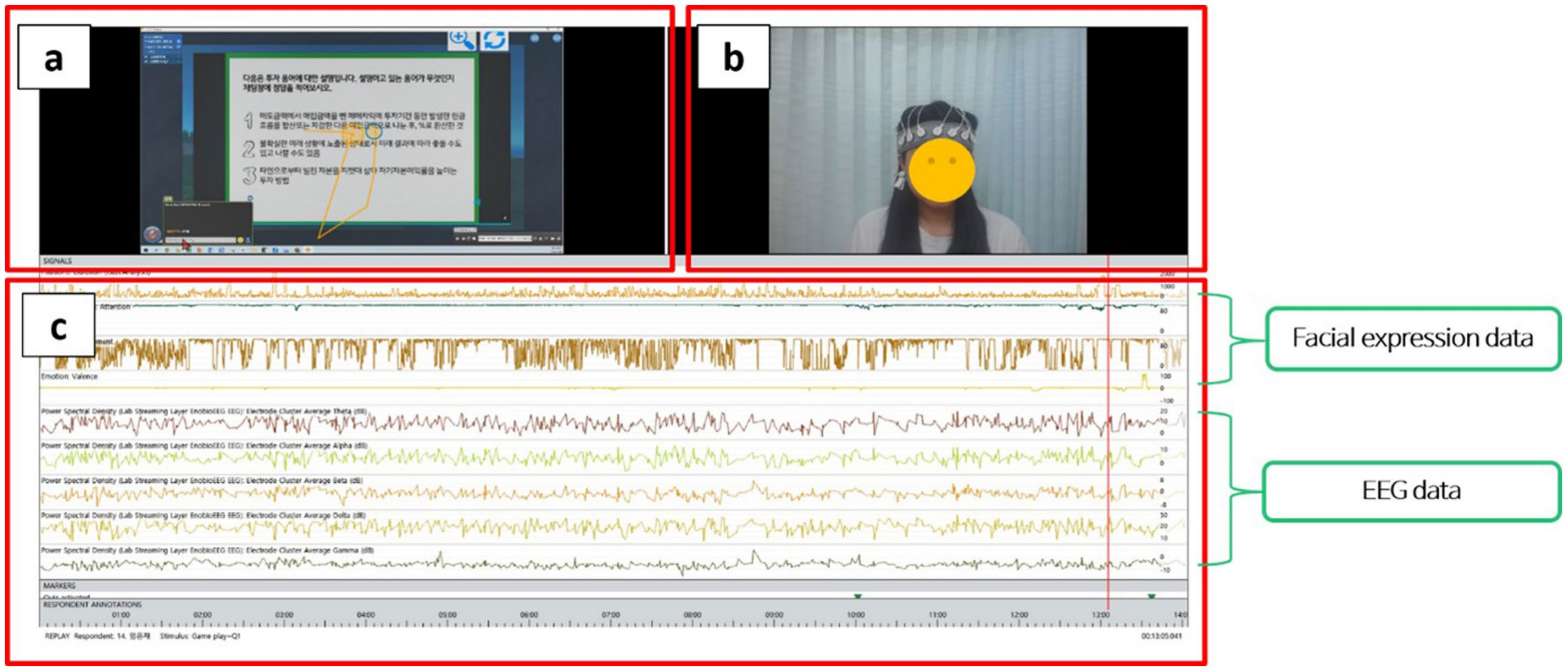
iMotions biometric research platform interface for multimodal recording and monitoring (**a**: gameplay scene; **b**: participant video for FEA; **c**: time-aligned multimodal signals).

The iMotions 9.3 Biometric Research Platform (iMotions, Copenhagen, Denmark) was used to integrate devices, synchronize multimodal streams (EEG and facial video), and log task events during gameplay. As shown in Figure 4, the interface provides the gameplay view, a live participant video feed for facial-expression analysis, and a time-aligned dashboard displaying facial metrics and EEG signals, enabling real-time monitoring of data quality and alignment across channels. Time-stamped event markers were used to delineate the three task periods (low, mid, high). Continuous EEG and facial-expression streams were segmented into task-specific time windows (hereafter, task segments) for feature extraction. For each participant and task segment, summary indices (FEA attention and engagement; EEG mental workload index and FAA) were extracted and exported for statistical analyses.

Data were analyzed using IBM SPSS Statistics (version 26.0). Self-reported mental effort was analyzed using a mixed-design ANOVA with SRL support condition (CS vs. MS vs. CG) as a between-subjects factor and task complexity (low vs. mid vs. high) as a within-subjects factor. Sphericity was assessed using Mauchly’s test; when violated, Greenhouse–Geisser corrections were applied. Significant effects were followed up with Bonferroni-adjusted pairwise comparisons.

For the facial-expression indices (attention, engagement) and EEG-derived indices (mental workload, FAA), linear mixed-effects models were fitted in IBM SPSS Statistics (v26; MIXED procedure) to account for repeated task measurements within participants. In each model, SRL support condition and task complexity were specified as fixed effects, and participant-specific random intercepts were included. Models were estimated using restricted maximum likelihood (REML). The within-subject residual covariance across the three task segments was modeled using a compound-symmetry structure. Fixed effects were evaluated using F tests with denominator degrees of freedom as computed in SPSS MIXED. When omnibus tests indicated significant main or interaction effects, follow-up comparisons were conducted with Bonferroni adjustment.

### 2.6 Experimental setting

**Figure 5:**
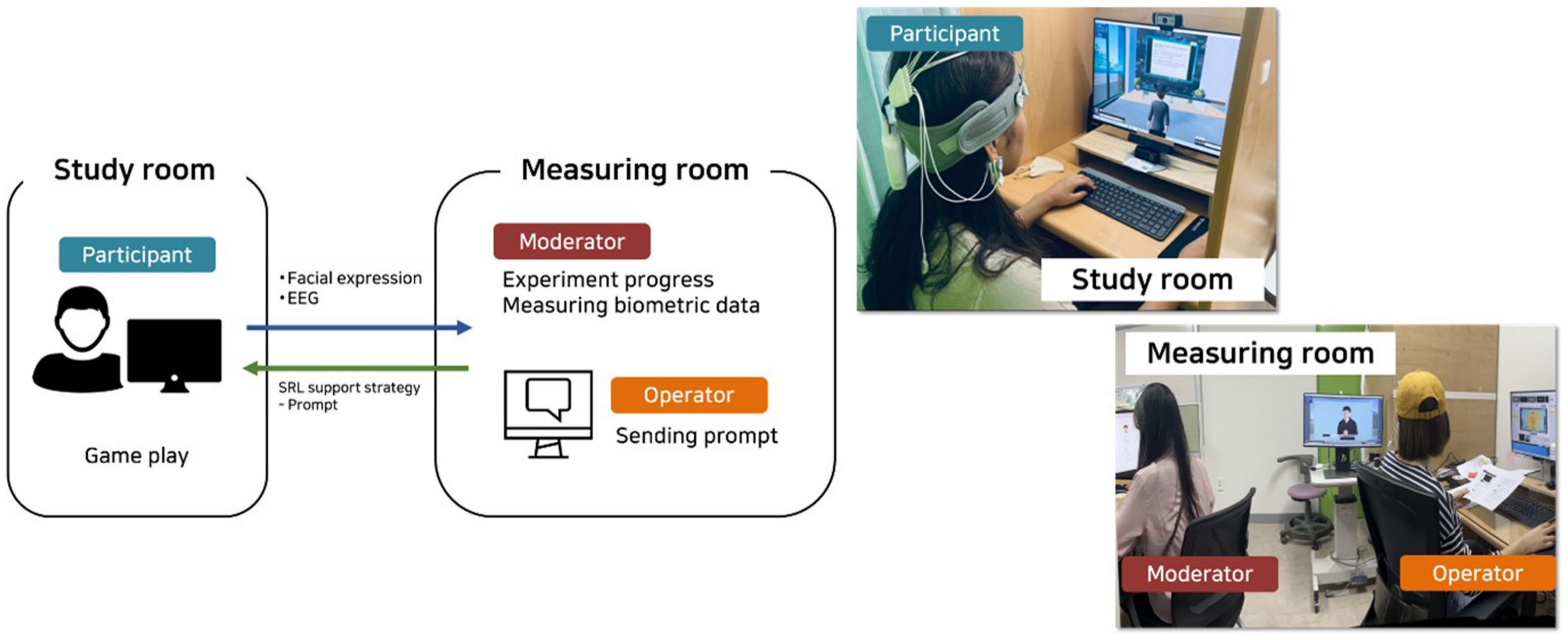
Experimental setting: study room and measurement room.

As shown in Figure 5, the experimental space consisted of a study room and a separate measurement room. In the study room, participants completed the learning activities on a monitor in a distraction-minimized booth. Participants were seated approximately 60 cm from a 16:10 monitor while EEG electrodes were applied and signal quality was verified using iMotions. Participants were instructed to minimize head movements to support stable facial video and EEG recording. In the adjacent measurement room, a moderator monitored task progress and recording quality. An operator delivered the pre-scripted SRL prompts within the MLE according to the assigned condition and the participant’s progression through the game.

## 3 Results

### 3.1 Cognitive loads

Self-reported mental effort was analyzed using a mixed-design ANOVA, with SRL support condition (CS vs. MS vs. CG) as a between-subjects factor and task complexity (low vs. mid vs. high) as a within-subjects factor. Mauchly’s test indicated that the sphericity assumption was met (*p* > 0.05). There was a significant main effect of SRL support condition on mental effort, *F*_(2, 39)_ = 4.43, *p* < 0.05, *η_p_*^2^ = 0.19. Scheffé *post-hoc* tests indicated that CG reported higher mental effort than CS (*p* < 0.05), suggesting a supportive trend of CS in mitigating perceived strain. Task complexity also showed a significant main effect, *F*_(2, 78)_ = 5.32, *p* < 0.01, *η_p_*^2^ = 0.12. Bonferroni-adjusted pairwise comparisons indicated that the mid-complexity task elicited greater mental effort than the high-complexity task (*p* < 0.01; Table 2). However, no interaction effect from the SRL support condition and task complexity level on mental effort was found.

**Table 2:** Descriptive statistics for cognitive load related outcomes by condition and task complexity (*N* = 42).

Outcome	Group	Low complexity	Mid-complexity	High complexity
		Mean (±SD)	Mean (±SD)	Mean (±SD)
Mental effort (Self-report)	CS (*n* = 13)	4.23 (±0.62)	4.31 (±0.71)	3.96 (±0.85)
	MS (*n* = 14)	4.30 (±0.48)	4.34 (±0.58)	4.21 (±0.54)
	CG (*n* = 15)	4.62 (±0.51)	4.83 (±0.31)	4.70 (±0.41)
Attention (FEA)	CS (*n* = 13)	97.27 (±2.37)	97.80 (±0.66)	96.55 (±2.68)
	MS (*n* = 14)	97.57 (±1.11)	97.64 (±0.82)	97.35 (±0.66)
	CG (*n* = 15)	97.71 (±0.93)	97.76 (±0.79)	96.95 (±1.62)
Mental workload (EEG)	CS (*n* = 13)	−0.18 (±0.21)	−0.17 (±0.17)	−0.22 (±0.19)
	MS (*n* = 14)	−0.22 (±0.16)	−0.14 (±0.13)	−0.18 (±0.18)
	CG (*n* = 15)	−0.26 (±0.30)	−0.20 (±0.33)	−0.27 (±0.30)

Behavioral (FEA-derived attention) and physiological (EEG beta/(alpha + theta) mental workload index) indicators were analyzed using linear mixed-effects models to account for repeated measurements across tasks. While these indicators showed significant fluctuations across task complexity levels, they remained relatively stable across SRL support conditions. For attention, task complexity showed a significant effect, *F*_(2, 78)_ = 6.52, *p* < 0.01 (goodness of fit: −2 RLL = 415.517; AIC = 421.517; BIC = 429.803). *Post hoc* comparisons indicated that attention was significantly lower during the high-complexity task than during the low-complexity task (*p* < 0.05) and the mid-complexity task (*p* < 0.01). For the EEG mental workload index, task complexity was also significant, *F*_(2, 77.99)_ = 6.62, *p* < 0.01 (goodness of fit: −2 RLL = −126.605; AIC = −120.605; BIC = −112.319). *Post hoc* comparisons indicated that EEG workload was higher in the mid-complexity task than in the low- and high-complexity tasks (*p*s < 0.01). In both models, neither the main effect of SRL support condition nor the SRL support condition × task complexity interaction reached statistical significance, indicating that the impact of SRL prompts on these physiological metrics was relatively subtle within the current experimental context.

### 3.2 Emotional engagement

Engagement (FEA-derived engagement metric) and frontal alpha asymmetry (FAA) were analyzed using LMM across the three task-complexity levels. For FEA-derived engagement, there was a significant main effect of task complexity, *F*_(2, 78)_ = 7.70, *p* < 0.01 (goodness of fit: −2 RLL = 957.559; AIC = 963.559; BIC = 971.846). Bonferroni-adjusted *post hoc* tests indicated that engagement was significantly higher during the mid-complexity task than during the low- and high-complexity tasks (*p*s < 0.01; Table 3).

**Table 3:** Descriptive statistics for affective engagement outcomes by condition and task complexity (*N* = 42).

Outcome	Group	Low complexity	Mid-complexity	High complexity
		Mean (±SD)	Mean (±SD)	Mean (±SD)
Engagement (FEA)	CS (*n* = 13)	35.62 (±20.64)	48.59 (±26.04)	35.11 (±21.36)
	MS (*n* = 14)	24.24 (±19.46)	30.61 (±16.35)	26.46 (±19.90)
	CG (*n* = 15)	25.57 (±25.61)	24.70 (±24.27)	26.44 (±24.64)
Frontal alpha asymmetry (EEG)	CS (*n* = 13)	−0.35 (±0.20)	−0.36 (±0.23)	−0.39 (±0.24)
	MS (*n* = 14)	−0.32 (±0.15)	−0.28 (±0.13)	−0.32 (±0.20)
	CG (*n* = 15)	−0.34 (±0.27)	−0.20 (±0.34)	−0.37 (±0.26)

**Figure 6:**
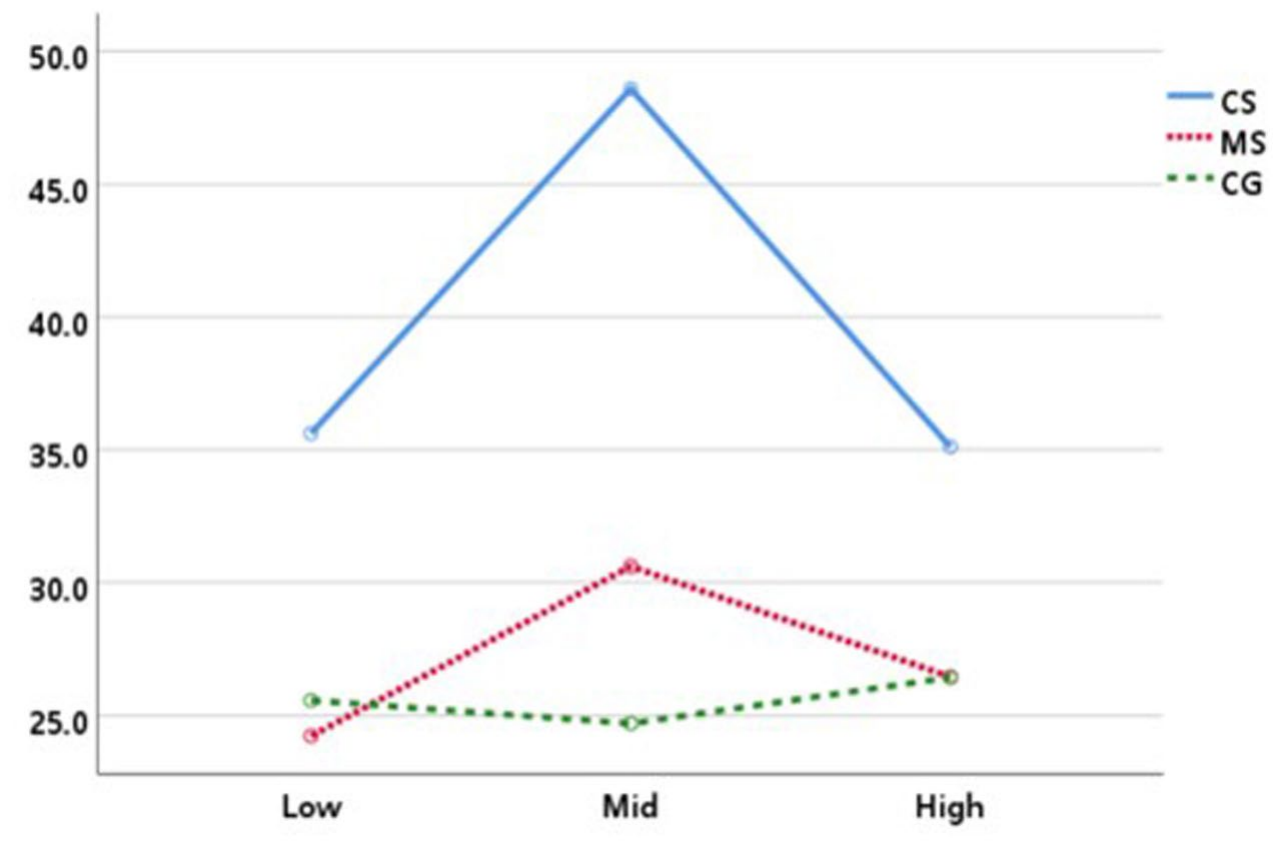
SRL support condition × task complexity interaction on FEA-derived engagement.

A significant SRL support condition × task complexity interaction was also observed, *F*_(4, 78)_ = 4.15, *p* < 0.01. Follow-up comparisons within the mid-complexity task indicated that engagement in CS was higher than in both MS and CG (Bonferroni-adjusted ps < 0.001; Figure 6). While this interaction suggests that CS prompts may have particularly facilitated engagement during the procedural task, it should be noted that the descriptive differences across conditions in the low and high complexity tasks were less pronounced. Descriptively, engagement in CS increased from low to mid complexity (low: *M* = 35.62, *SD* = 20.64; mid: *M* = 48.59, *SD* = 26.04) and decreased at high complexity (*M* = 35.11, *SD* = 21.36), whereas MS and CG showed smaller changes across task-complexity levels (Table 3).

For FAA, there was a significant main effect of task complexity, *F*_(2, 78)_ = 4.69, *p* < 0.05 (goodness of fit: −2 RLL = −53.006; AIC = −47.006; BIC = −38.719). Post hoc comparisons indicated that FAA was higher during the mid-complexity task than during the high-complexity task (*p* < 0.05). The main effect of SRL support condition and the SRL support × task complexity interaction were not significant. However, the main effect of SRL support condition and the SRL support × task complexity interaction for FAA were not significant, reflecting a degree of physiological stability across the intervention groups.

## 4 Discussion

In this study, we investigated the interplay between SRL support strategies and task complexity in a MLE using a multimodal approach. By analyzing subjective, physiological, and behavioral data, we examined how cognitive and motivational regulation supports influence learners’ cognitive load and emotional engagement across tasks of varying complexity. The results provide empirical evidence for the utility of multimodal triangulation in capturing the nuanced dynamics of learner experience in immersive settings.

### 4.1 Main findings

The results revealed distinct patterns across measures, highlighting the complex relationship between instructional support and learner experience. Four key findings warrant discussion.

First, the results suggest that SRL support may foster instructional efficiency by mitigating the overall cognitive load. Contrary to the concern that pop-up prompts in immersive environments might act as intrusive interruptions (Wang 2025a; Wang 2025b), the Control Group (CG) reported significantly higher mental effort than the Cognitive Support (CS) group. From a CLT perspective, this finding suggests a successful scaffolding effect. The CG participants, lacking external guidance, likely bore a heavier burden of intrinsic regulation—the executive demand of planning and monitoring one’s own learning process—which manifested as higher subjective strain (Sweller et al., 2011). Conversely, the SRL prompts in the CS condition may have functioned as an external regulator, potentially offloading part of these executive demands and allowing learners to allocate their cognitive resources more effectively (Seufert, 2018). While the current measures do not empirically dissociate specific types of cognitive load, the observed reduction in subjective mental effort may indicate more efficient management of cognitive resources under the CS condition. The relative stability of physiological arousal and behavioral engagement across conditions further suggests that these scaffolds may have helped learners manage tasks with less perceived strain (Paas and van Merriënboer, 1993). However, whether this reflects optimized cognitive efficiency or merely a reduction in perceived task demands remains unclear and requires further empirical investigation. Accordingly, the present findings provide an initial indication of potential cognitive relief, although the underlying mechanism—and its direct impact on learning outcomes—remains to be empirically tested in future research (Leppink et al., 2013).

Second, task complexity effects were robust but non-monotonic, driven by working memory intensity rather than mere information volume. Both the EEG-derived workload index and FEA-derived attention peaked during the mid-complexity task (mental arithmetic) rather than the high-complexity task (open-ended problem solving). This convergence indicates that in MLEs, the nature of element interactivity dictates physiological arousal more than the total amount of content (Van Merriënboer and Sweller, 2010). The mental arithmetic task required the continuous, active maintenance of information in working memory without external visual cues, creating a high-intensity cognitive demand. While this peak arousal reflects the inherent nature of mental calculation tasks, it confirms that in MLEs, complexity is experienced as the immediacy of processing rather than just the volume of information. This finding resonates with the transient information effect, suggesting that tasks requiring immediate internal processing trigger sharper physiological responses than those supported by external representations (Leahy and Sweller, 2011). Furthermore, participants may have possessed lower relative expertise in mental arithmetic compared to conceptual understanding or open-ended problem-solving tasks. The requirement for continuous working memory updates, potentially coupled with varying levels of arithmetic fluency among participants, could have independently intensified the observed cognitive load and emotional activation, beyond the intended level of complexity. Also, this non-monotonic pattern may reflect an inverted U-shaped relationship between task difficulty and engagement. The mid-complexity task may have provided an ‘optimal match’ to the participants’ prior knowledge, triggering maximal resource allocation, whereas the high-complexity task might have exceeded their perceived capabilities, leading to a state of cognitive overload and subsequent disengagement (Seufert, 2018; Ten et al., 2025).

Third, a critical dissociation emerged between subjective effort and emotional engagement, pointing to cognitive-emotional optimization. While the CS group reported lower subjective mental effort, they exhibited the highest emotional engagement (FEA) specifically during the most cognitively demanding (mid-complexity) task. This interaction implies that cognitive SRL supports did not simply make the task easier by lowering standards; rather, they fostered a state of productive immersion. According to Control-Value Theory, clear guidance (scaffolding) can increase learners’ sense of control, thereby reducing anxiety (extraneous load) and promoting positive activating emotions like enjoyment or focused engagement (Pekrun, 2006). The CS group’s ability to maintain high emotional engagement with reduced subjective strain suggests that SRL scaffolds enabled them to bypass inefficient frustration and channel their energy into productive task execution. This supports the argument that multimodal data can disentangle perceived struggle from productive engagement, demonstrating that SRL supports help learners manage high-load tasks with greater emotional resilience and less subjective exhaustion (Azevedo, 2015). Furthermore, considering the fixed experimental sequence, this peak in the mid-level task may also represent the participants’ maximal cognitive investment before cumulative fatigue or sensory overload affected their involvement in the final task. This suggests that the observed engagement was a combined product of both the internal scaffolding provided by SRL supports and the temporal dynamics of the learners’ cognitive stamina throughout the session.

Fourth, the lack of significant effects for MS suggests a potential boundary for emotional scaffolding in highly immersive environments. One interpretation is that the intense situational interest and novelty inherent in the metaverse maintained participants’ motivation at a relatively high level from the outset, rendering additional external prompts less impactful. Importantly, the observed inverted U-shaped effect—where engagement peaked at mid-level task complexity—indicates that participants may have experienced an optimal balance between challenge and skill. In this condition, participants’ need for competence was likely satisfied through successful task completion, thereby reinforcing intrinsic motivation (Seufert et al., 2017). Consequently, the MS prompts may have failed to show incremental effects not merely because the medium itself was engaging, but because learners were already operating at a high level of engagement induced by the task structure. These perspectives are not mutually exclusive; rather, they suggest that in Metaverse-based instruction, the design of emotional scaffolds should be more nuanced. Motivational support may be less effective when situational interest is high or when perceived competence is already optimal, but it may become more critical when the novelty of the environment wanes or when task complexity exceeds the learner’s current capacity.

### 4.2 Implications

The findings of this study offer several theoretical and practical implications for designing and evaluating educational experiences in MLEs. First, from an instructional design perspective, the results suggest that SRL prompts can function as effective tools for reducing overall cognitive strain. Although interactive features in the Metaverse are often scrutinized for potential distraction, our study suggests that when prompts function as regulatory scaffolds, they significantly improve instructional efficiency (Paas and van Merriënboer, 1993). The fact that the CS group reported lower mental effort while maintaining high emotional engagement suggests that the supports successfully absorbed the “regulatory overhead”—the cognitive cost of self-management. Therefore, designers of MLEs should utilize explicit guidance interfaces not merely as reminders, but as external regulators that optimize the trade-off between cognitive effort and emotional involvement.

Second, this study highlights the necessity of distinguishing between “informational complexity” and “working memory intensity” when sequencing tasks. The robust physiological and behavioral peaks observed during the mid-complexity task indicate that tasks requiring immediate, internal information maintenance trigger stronger arousal than text-heavy tasks. This implies that in immersive environments, cognitive load is driven less by the complexity of the content and more by the immediacy of the processing demands. Educators should be aware that “short but intense” tasks (like mental calculation or timed decision-making) serve as stronger drivers of physiological arousal than longer, passive tasks. Consequently, curriculum sequencing in the Metaverse should balance these high-intensity modalities with self-paced activities to prevent cognitive overload and fatigue (Makransky and Petersen, 2021).

Third, methodologically, this study validates the necessity of a multidimensional measurement approach that integrates process and appraisal. Traditional triangulation often seeks convergence across measures. However, our findings highlight the value of complementarity: EEG and FEA captured the moment-to-moment processing costs (process), while self-reports captured the learner’s post-hoc evaluation of effort (appraisal). The dissociation between these two—where physiological arousal was maintained (or heightened) while perceived effort dropped—reveals that SRL supports alter how learners experience the load, even if the biological cost remains comparable. Therefore, future measurement frameworks should explicitly model these channels not as redundant validators, but as distinct layers of the learning experience: physiological cost (Process) vs. perceived strain (Product).

### 4.3 Limitations

Several limitations should be noted when interpreting these findings. First, the sample size was relatively modest (N = 42), which may have limited the statistical power to detect smaller interaction effects between SRL conditions and task complexity. Although the sample size is relatively modest due to the intensive nature of multimodal data collection, the study employed a repeated-measures design (three tasks per participant) to maximize statistical power for detecting within-subject complexity effects. To mitigate potential power issues, we also focused on interpreting effect sizes (partial eta squared) alongside statistical significance. However, future studies with larger cohorts are needed to generalize the findings to broader populations.

Second, the order effect can be affected in this study because the learning tasks were administered in a fixed sequence and differed in the tasks. While this design reflected Bloom’s taxonomy, it introduces qualitatively different cognitive processing demands inherent in each task type. However, the fact that physiological arousal peaked at the mid-complexity task (rather than the final high-complexity task) suggests that the specific demands of the mental arithmetic task likely outweighed simple fatigue effects. Nevertheless, we acknowledge that these task-specific characteristics may independently influence cognitive load and emotional engagement, making it difficult to isolate the effect of complexity per se. Therefore, future research should employ counterbalanced designs or consistent task formats within a single task type to isolate the effects of complexity more precisely.

Third, while we tentatively interpreted the dissociation between lowered subjective effort and sustained emotional engagement as evidence of cognitive efficiency, this conclusion relies on indirect proxies. The absence of direct learning outcome measures (e.g., post-test scores or knowledge retention levels) limits the interpretation of the study’s educational effectiveness. Although we utilized FEA and EEG as indicators of processing intensity, we did not definitively confirm whether higher workload or engagement translated into superior learning achievement. Without linking process indicators to instructional efficiency, the results remain focused on the how of learning rather than its success. To address this limitation, future research should integrate standardized knowledge assessments and performance metrics—such as pre- and post-test accuracy, task completion time, and long-term retention scores—to determine the direct relationship between neuro-behavioral processing and educational gains. By triangulating real-time sensor data with these objective performance indicators, future work will be able to confirm whether cognitive efficiency and emotional engagement during the process truly correlate with optimal learning outcomes (Wu et al., 2014).

Fourth, while this study prioritized physiological and behavioral indicators to capture the transient dynamics of emotional engagement, the absence of self-report measures remains a limitation. Although biological proxies can minimize retrospective bias, future research should incorporate validated post-task questionnaires to enable a more robust triangulation of engagement across multiple data sources. Such an integration would help contextualize the subjective meaning of the physiological states captured through multimodal data and provide additional insight into how moment-to-moment motivational dynamics during metaverse interactions relate to broader patterns of learner engagement. To address this gap, future studies should examine the relationship between real-time neurobehavioral markers and retrospective self-report measures, thereby enabling a more comprehensive understanding of motivational processes in immersive learning environments (Wang 2025a; Wang 2025b).

Finally, the EEG analysis was constrained by the hardware specifications (60 Hz sampling rate) of the integrated headset used. While this device allowed for high ecological validity and unobtrusive measurement, it limited the analysis to lower frequency bands and basic workload indices. Future studies aiming for finer-grained neural correlates of cognitive load should consider using higher-density EEG systems with faster sampling rates to capture broader spectral features.

## 5 Conclusion

This study set out to disentangle the complex dynamics of learner experience in the Metaverse by applying a multimodal lens to the interaction between SRL supports and task complexity. Our investigation leads to a critical insight: in immersive learning environments, optimizing the balance between cognitive strain and emotional involvement is paramount. The most significant theoretical contribution of this work is the empirical demonstration of cognitive-emotional optimization within the SRL context. By uncovering the dissociation between subjective mental effort and emotional engagement, we observed that effective SRL supports enabled learners to maintain high emotional engagement with reduced subjective strain. Rather than implying that learners were disengaged, the combination of lowered mental effort and heightened emotional engagement suggests a state of productive immersion. SRL supports helped learners bypass unnecessary regulatory overhead, allowing them to remain emotionally connected to the learning experience without being overwhelmed by cognitive load. This challenges the assumption that high perceived effort is a prerequisite for deep engagement, suggesting instead that ideal immersive learning should be designed to maximize emotional involvement while minimizing subjective cognitive cost.

Methodologically, this study serves as a cautionary tale against single-channel assessment in EdTech research. The divergence we observed—where learners felt less burdened yet responded with greater emotional intensity—would have remained invisible to traditional self-report measures alone. This finding establishes multimodal triangulation not merely as a supplementary option, but as a prerequisite for validly interpreting the black box of learner experience in high-load virtual environments. In sum, as immersive technologies become increasingly integrated into educational landscapes, the design focus must shift from simply delivering content to orchestrating the learner’s cognitive economy and emotional resonance. Future research must continue to bridge the gap between what learners say they feel (subjective load) and how their bodies expressively respond (emotional engagement), ensuring that we build virtual learning spaces that are not only technologically immersive but also psychologically sustainable.

## Data Availability

The datasets presented in this article are not readily available because they contain sensitive biometric data, including facial expressions and EEG, which are protected under IRB confidentiality protocols to ensure participant anonymity. Requests to access the datasets should be directed to Eunbyul Yang, e.yang@jejunu.ac.kr.
